# Three Years of Idiopathic Chylous Ascites: Splenic Marginal Zone Lymphoma Behind the Mask

**DOI:** 10.7759/cureus.101496

**Published:** 2026-01-13

**Authors:** Rova Malala Fandresena Randrianarisoa, Scholastique Ngo Souck M, Mostefa Koroghli, Ismail Meziane, Catherine Letrillard

**Affiliations:** 1 Internal Medicine, Chauny Hospital, Chauny, FRA; 2 Medical-Surgical Unit, Chauny Hospital, Chauny, FRA

**Keywords:** b-cell lymphoma, chylous ascites, flow cytometry, splenic marginal zone lymphoma, splenomegaly

## Abstract

Chylous ascites is rare in adults and is most commonly caused by lymphatic obstruction due to non-Hodgkin B-cell lymphoma. Splenic marginal zone lymphoma (SMZL) is an uncommon etiology. We report the case of an 89-year-old man with a three-year history of refractory chylous ascites initially considered of unknown origin. Clinical examination revealed significant ascites and splenomegaly. PET imaging demonstrated hypermetabolic lymphadenopathy, and bone marrow biopsy revealed infiltration by a CD5-/CD10- B-cell lymphoma consistent with SMZL. Analysis of ascitic fluid showed a predominance of T lymphocytes without a detectable B-cell clone. This case illustrates that SMZL, although rare, should be included in the differential diagnosis of persistent chylous ascites. A comprehensive diagnostic approach combining imaging, bone marrow biopsy, and flow cytometry is essential to establish a timely diagnosis and guide appropriate management, especially in elderly patients.

## Introduction

Chylous ascites accounts for less than 1% of all ascites cases. It most commonly results from lymphatic obstruction or rupture due to a tumor, infection, trauma, or surgery. In adults, non-Hodgkin B-cell lymphomas are among the primary malignant causes of chylous ascites, whereas other types of lymphoma, particularly indolent forms, are much rarer [1]. SMZL is a rare, indolent B-cell lymphoma, accounting for less than 2% of non-Hodgkin lymphomas. It typically presents as isolated splenomegaly and may be associated with cytopenias and mild lymphocytosis [2]. Chylous ascites is an exceptionally rare presentation of SMZL [3]. Here, we report a case of refractory chylous ascites that developed over several years and was ultimately diagnosed as SMZL.

## Case presentation

An 89-year-old man had been monitored for three years due to refractory ascites. During this period, he underwent repeated paracenteses, which revealed chylous ascites. Initial evaluations showed a monoclonal IgG kappa peak, while Bence Jones protein testing was negative. A thoracoabdominal-pelvic CT scan revealed the presence of ascites and splenomegaly. In the absence of further investigation, the condition was considered idiopathic ascites. His medical history included hypertension, dyslipidemia, and stage 3B chronic kidney disease.

In the context of his current history, he was admitted to our department for the first time due to a deterioration in his general condition. His hemodynamic parameters were stable. Clinical examination revealed significant ascites and splenomegaly. The patient reported no joint pain. There was no peripheral lymphadenopathy, and the remainder of the examination was unremarkable.

Blood tests revealed isolated, non-regenerative, normocytic anemia with a normal lymphocyte count. Renal function remained stable, with a serum creatinine of 182 µmol/L. Serum calcium was elevated at 3.0 mmol/L, while parathyroid hormone levels were normal. Liver function tests, total protein, and TSH were within normal limits. Serology and urine bacteriology were negative. Ferritin was 141 ng/mL, and beta-2-microglobulin was 15.3 mg/L. The kappa/lambda free light chain ratio was 0.967. Serum immunoglobulin levels were normal.

**Figure 1:**
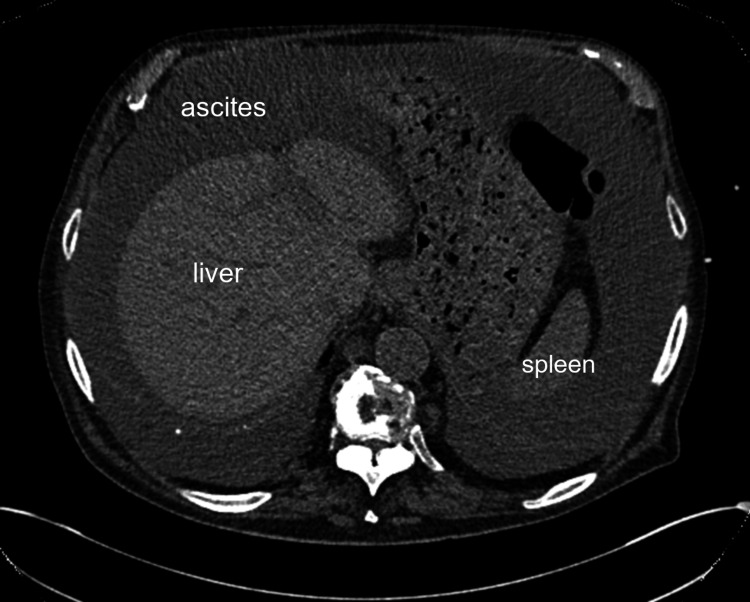
Non-contrast thoracoabdominal-pelvic CT scan (axial view) showing abundant ascites

A thoracoabdominal-pelvic CT scan revealed significant ascites, splenomegaly, and mesenteric lymphadenopathy (Figure 1). PET imaging showed splenomegaly and multiple hypermetabolic supra- and subdiaphragmatic lymph nodes.

Bone marrow biopsy revealed increased and heterogeneous marrow cellularity, infiltrated by a small B-cell non-Hodgkin lymphoma involving 20% of the marrow, which was difficult to classify. Flow cytometry demonstrated 22% abnormal monotypic lambda B lymphocytes with high intensity. The Matutes score was 2. The immunophenotype was consistent with SMZL (Table 1).

**Table 1:** Immunophenotypic profile of bone marrow B cells

	Results
Positive	CD20
	CD79a
Negative	CD5
	CD10
	CD43
	CD200
	CD23
	CD 138
	Cyclin D1

Analysis of the ascitic fluid revealed a chylous effusion with a triglyceride level of 3.8 g/L, a protein concentration of 48 g/L, and 817 cells/mm³, 85% of which were lymphocytes. Flow cytometry showed that 61% of the cells were lymphocytes, predominantly CD4+ T cells, with no detectable surface light chains on B lymphocytes.

The diagnosis of SMZL was confirmed based on intrasinusoidal infiltration, bone marrow immunophenotyping, and flow cytometry of the ascitic fluid. Treatment with rituximab-bendamustine was planned; however, follow-up at our center was not possible because, after the diagnosis was established, he was transferred to a specialized tertiary hospital for subsequent management.

## Discussion

SMZL is a slow-growing, indolent lymphoma that usually presents with isolated splenomegaly. Unlike other malignant hematologic disorders, it typically does not cause massive retroperitoneal involvement or mesenteric lymphatic compression, which explains why chylous ascites is rare [2,4]. In our patient, disseminated hypermetabolic supra- and subdiaphragmatic lymph nodes were observed. The ascites could be attributed to abdominal lymphatic compression. We found only one comparable case in the literature: in 2013, Campbell-Silva et al. reported a case of SMZL complicated by splenic rupture and chylous ascites in a 51-year-old man [3]. Although uncommon, this observation suggests that SMZL should be considered in the differential diagnosis of chylous ascites.

In our case, the ascitic fluid was predominantly composed of CD4+ T lymphocytes, with no detectable B-cell clone. This predominance of T cells likely reflects a reactive immune response within the peritoneal environment, rather than a direct infiltration of the fluid by neoplastic B cells. The absence of surface light chain restriction on B lymphocytes does not exclude the presence of a B-cell lymphoma, as the neoplastic clone may be present at low frequency or confined to other compartments. Therefore, a reactive T-cell profile can mask a neoplastic B-cell population, and routine cytology alone is insufficient to rule out a B-cell hematologic malignancy. Comprehensive flow cytometry, including B-cell clonality assessment and light chain analysis, remains essential to detect lymphoma, particularly in atypical presentations such as chylous or T-cell-predominant effusions [5,6].

The bone marrow biopsy was decisive. A monotypic lambda B-cell population was identified, with the immunophenotype CD20+, CD79a+, CD23-, CD5-, cyclin D1-, and CD138-, consistent with SMZL. This finding allowed for a definitive diagnosis without the need for splenectomy. Such a diagnostic approach offers a clear advantage in elderly patients and aligns with recent recommendations for the management of SMZL [7,8].

The treatment of SMZL depends on disease severity and includes several options: observation, splenectomy, immunotherapy, or immunochemotherapy. A combination of rituximab and bendamustine is often used for symptomatic or extensive disease, offering high response rates and prolonged remissions [7,8]. Ascites typically resolves following effective disease management. In the case reported by Campbell-Silva et al., splenectomy was required due to splenic rupture [3].

This case underscores the importance of a thorough evaluation before labeling ascites as idiopathic, as rare causes such as indolent lymphoma may be missed. It highlights the value of flow cytometry on ascitic fluid to detect neoplastic B-cell clones, even when cytology appears reactive, and the need to consider SMZL in cases of persistent chylous ascites. Finally, it demonstrates that a combination of imaging and bone marrow biopsy can establish a diagnosis of SMZL without splenectomy, a strategy particularly appropriate for elderly or frail patients.

## Conclusions

This case describes an unusual presentation of SMZL, revealed by refractory chylous ascites initially classified as idiopathic. Although rare, it underscores the importance of considering SMZL in the differential diagnosis of persistent chylous ascites. An integrated approach combining imaging, flow cytometry, and histopathology is essential for timely diagnosis and appropriate management, particularly in elderly patients.
